# P-1230. Tesamorelin (EGRIFTA WR and EGRIFTA®) Formulations: Bioequivalence in Healthy Participants

**DOI:** 10.1093/ofid/ofaf695.1422

**Published:** 2026-01-11

**Authors:** Christian Caldji, Christian Marsolais, Eric Sicard, Marilyn de Chantal, Kaitlin Anstett

**Affiliations:** Theratechnologies, Montreal, QC, Canada; Theratechnologies, Montreal, QC, Canada; AltaSciences, Montreal, Quebec, Canada; Theratechnologies Inc., Montréal, Quebec, Canada; Theratechnologies Inc., Montréal, Quebec, Canada

## Abstract

**Background:**

Tesamorelin (*EGRIFTA*^®^) is a synthetic analog of the growth hormone releasing hormone, indicated for the treatment of excess abdominal fat in adults with HIV. The original formulation is 2 mL injected subcutaneously (SC) into abdominal skin with a 1 mg/mL formulation. A study was conducted to evaluate the bioequivalence, safety and tolerability of a more concentrated formulation with a smaller volume of administration, 0.16 ml per dose to tesamorelin (*EGRIFTA* WR^TM^).

**Figure:**
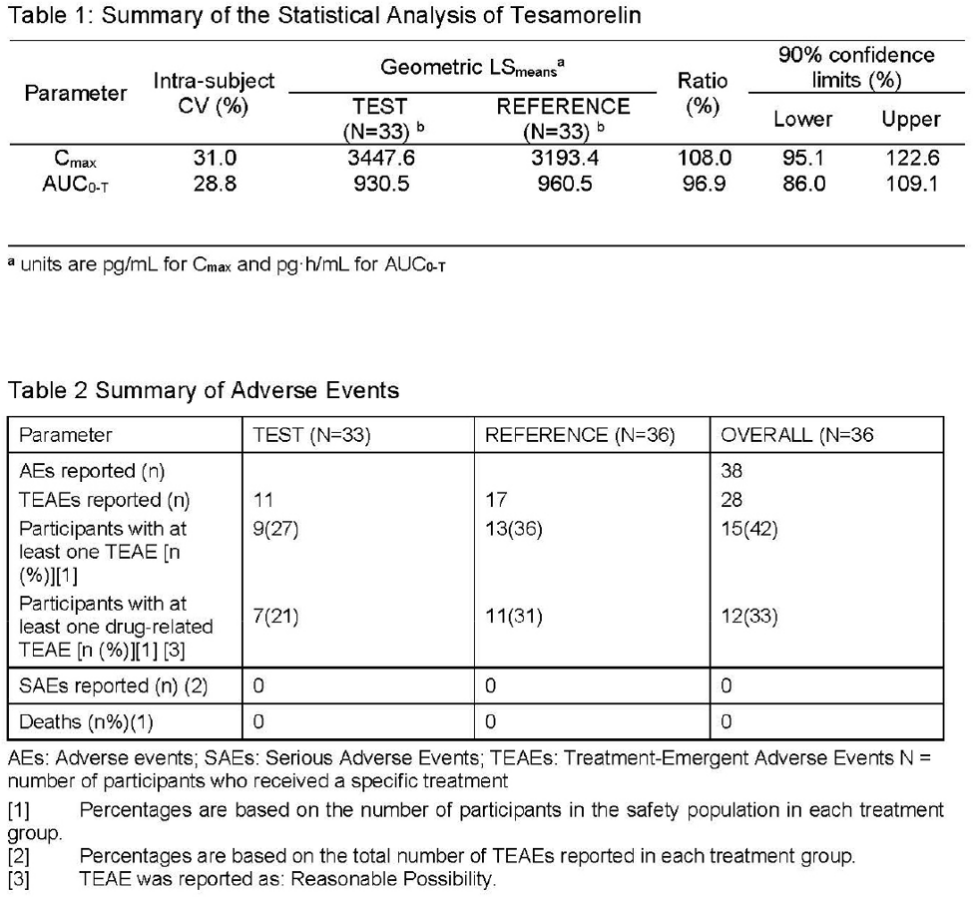

**Methods:**

This phase I study was a single center, randomized, blinded, 2-treatment, 2-period, 2-sequence, crossover, single dose design, in which 36 healthy adult participants received one of the study treatments during each study period. Participants received each of the following doses of tesamorelin SC:

Test: 1.28 mg (0.16 mL)

Reference: 2 mg (2 mL)

**Results:**

The PK results demonstrate that the geometric LSmean ratios of C_max_ and AUC_0-t_ of tesamorelin were 108.0% and 96.9%, respectively (Table 1). For AUC_0-t_, the corresponding 90% CIs were included within the range of 80.0% to 125.0%. No statistically significant treatment, sequence or period effects were observed for the ln-transformed C_max_ and AUC_0-t_ data. The results of this study indicate that bioequivalence criteria were met. The incidence of drug-related Treatment Emergent Adverse Events (TEAEs) was of 21% following administration of the Test and 31% following administration of the Reference. The TEAE experienced most commonly in this study was injection site pain, reported 12% after administration of the Test and 19% after administration of the Reference.

**Conclusion:**

The results presented here show that the criteria used to assess bioequivalence between the Test and Reference formulations were all fulfilled. The Test to Reference ratio of geometric LS_means_ for the C_max_ was within the acceptance range of 80.0 to 125.0%. Furthermore, the ratio and 90% CI for the Test to Reference ratio of geometric LS_means_ for AUC_0-t_ were also within the acceptance range of 80.0 to 125.0%. The Test formulation is judged to be bioequivalent to the Reference formulation. Overall, both formulations of tesamorelin tested were generally safe (with fewer adverse events in the new formulation) and well tolerated by the participants included in this study.

**Disclosures:**

Christian Caldji, PhD, Theratechnologies: Employee Christian Marsolais, PhD, Theratechnologies: Employee Marilyn de Chantal, Ph.D., Theratechnologies: Employee Kaitlin Anstett, PhD, Theratechnologies: Employee

